# THE IMPACT OF SUBJECTIVE WELL-BEING ON LONGEVITY AMONG OLDER ADULTS

**DOI:** 10.1093/geroni/igz038.1147

**Published:** 2019-11-08

**Authors:** Sylvia Y Wang, Giyeon Kim, Ansley Gilpin

**Affiliations:** 1 The University of Alabama, Tuscaloosa, Alabama, United States; 2 Chung-Ang University, Seoul, Korea, Republic of

## Abstract

Objectives: People tend to believe happier people live longer. However, relatively few empirical studies have examined the influence of subjective well-being (SWB) on longevity among older adults. Thus, our study investigated the impact of SWB on longevity among older adult using national representative longitudinal data in the U.S. Methods: Drawn from the National Health and Aging Trends Study, 6,757 older adults aged 65 or older with completed information of SWB from 2011 were selected and followed until 2017 annually. The Kaplan-Meier estimator was used to estimate the survival time between different levels of SWB without covariates. In addition, the Cox Proportional Hazards Model was used to investigate the impact of SWB on longevity while adjusting the influences of covariates. Results: We found that a higher level of SWB predicted longer survival times among older adults. The impact of SWB on survival times remained to be significant, but weaker, after adjusting the influences of age, educational attainment, household income, gender, marital status, number of health insurances, self-rated health, chronical medical illness, and mental health. Conclusion: Findings suggest that happier older adults live longer. Recognizing the importance of SWB on longevity, healthcare providers should develop programs promoting higher SWB to prolong life for older adults.

